# Ferritin levels in the cerebrospinal fluid predict Alzheimer's disease outcomes and are regulated by APOE

**DOI:** 10.1038/ncomms7760

**Published:** 2015-05-19

**Authors:** Scott Ayton, Noel G. Faux, Ashley I. Bush, Alzheimer's Disease Neuroimaging Initiative, Michael W. Weiner, Paul Aisen, Ronald Petersen, Clifford R. Jack Jr., William Jagust, John Q. Trojanowki, Arthur W. Toga, Laurel Beckett, Robert C. Green, Andrew J. Saykin, John Morris, Leslie M. Shaw, Zaven Khachaturian, Greg Sorensen, Lew Kuller, Marc Raichle, Steven Paul, Peter Davies, Howard Fillit, Franz Hefti, Davie Holtzman, M. Marcel Mesulam, William Potter, Peter Snyder, Adam Schwartz, Tom Montine, Ronald G. Thomas, Michael Donohue, Sarah Walter, Devon Gessert, Tamie Sather, Gus Jiminez, Danielle Harvey, Matthew Bernstein, Nick Fox, Paul Thompson, Norbert Schuff, Bret Borowski, Jeff Gunter, Matt Senjem, Prashanthi Vemuri, David Jones, Kejal Kantarci, Chad Ward, Robert A. Koeppe, Norm Foster, Eric M. Reiman, Kewei Chen, Chet Mathis, Susan Landau, Nigel J. Cairns, Erin Householder, Lisa Taylor-Reinwald, Virginia Lee, Magdalena Korecka, Michal Figurski, Karen Crawford, Scott Neu, Tatiana M. Foroud, Steven Potkin, Li Shen, Kelley Faber, Sungeun Kim, Kwangsik Nho, Leon Thal, Neil Buckholtz, Marylyn Albert, Richard Frank, John Hsiao, Jeffrey Kaye, Joseph Quinn, Betty Lind, Raina Carter, Sara Dolen, Lon S. Schneider, Sonia Pawluczyk, Mauricio Beccera, Liberty Teodoro, Bryan M. Spann, James Brewer, Helen Vanderswag, Adam Fleisher, Judith L. Heidebrink, Joanne L. Lord, Sara S. Mason, Colleen S. Albers, David Knopman, Kris Johnson, Rachelle S. Doody, Javier Villanueva-Meyer, Munir Chowdhury, Susan Rountree, Mimi Dang, Yaakov Stern, Lawrence S. Honig, Karen L. Bell, Beau Ances, Maria Carroll, Sue Leon, Mark A. Mintun, Stacy Schneider, Angela Oliver, Daniel Marson, Randall Griffith, David Clark, David Geldmacher, John Brockington, Erik Roberson, Hillel Grossman, Effie Mitsis, Leyla deToledo-Morrell, Raj C. Shah, Ranjan Duara, Daniel Varon, Maria T. Greig, Peggy Roberts, Marilyn Albert, Chiadi Onyike, Daniel D'Agostino II, Stephanie Kielb, James E. Galvin, Brittany Cerbone, Christina A. Michel, Henry Rusinek, Mony J de Leon, Lidia Glodzik, Susan De Santi, P. Murali Doraiswamy, Jeffrey R. Petrella, Terence Z. Wong, Steven E. Arnold, Jason H. Karlawish, David Wolk, Charles D. Smith, Greg Jicha, Peter Hardy, Partha Sinha, Elizabeth Oates, Gary Conrad, Oscar L. Lopez, MaryAnn Oakley, Donna M. Simpson, Anton P. Porsteinsson, Bonnie S. Goldstein, Kim Martin, Kelly M. Makino, M. Saleem Ismail, Connie Brand, Ruth A. Mulnard, Gaby Thai, Catherine Mc-Adams-Ortiz, Kyle Womack, Dana Mathews, Mary Quiceno, Ramon Diaz-Arrastia, Richard King, Myron Weiner, Kristen Martin-Cook, Michael DeVous, Allan I. Levey, James J. Lah, Janet S. Cellar, Jeffrey M. Burns, Heather S. Anderson, Russell H. Swerdlow, Liana Apostolova, Kathleen Tingus, Ellen Woo, Daniel H.S. Silverman, Po H. Lu, George Bartzokis, Neill R Graff-Radford, Francine Parfitt, Tracy Kendall, Heather Johnson, Martin R. Farlow, Ann Marie Hake, Brandy R. Matthews, Scott Herring, Cynthia Hunt, Christopher H. van Dyck, Richard E. Carson, Martha G. MacAvoy, Howard Chertkow, Howard Bergman, Chris Hosein, Sandra Black, Bojana Stefanovic, Curtis Caldwell, Ging-Yuek Robin Hsiung, Howard Feldman, Benita Mudge, Michele Assaly, Andrew Kertesz, John Rogers, Charles Bernick, Donna Munic, Diana Kerwin, Marek-Marsel Mesulam, Kristine Lipowski, Chuang-Kuo Wu, Nancy Johnson, Carl Sadowsky, Walter Martinez, Teresa Villena, Raymond Scott Turner, Kathleen Johnson, Brigid Reynolds, Reisa A. Sperling, Keith A. Johnson, Gad Marshall, Meghan Frey, Barton Lane, Allyson Rosen, Jared Tinklenberg, Marwan N. Sabbagh, Christine M. Belden, Sandra A. Jacobson, Sherye A. Sirrel, Neil Kowall, Ronald Killiany, Andrew E. Budson, Alexander Norbash, Patricia Lynn Johnson, Joanne Allard, Alan Lerner, Paula Ogrocki, Leon Hudson, Evan Fletcher, Owen Carmichael, John Olichney, Charles DeCarli, Smita Kittur, Michael Borrie, T-Y Lee, Rob Bartha, Sterling Johnson, Sanjay Asthana, Cynthia M. Carlsson, Steven G. Potkin, Adrian Preda, Dana Nguyen, Pierre Tariot, Stephanie Reeder, Vernice Bates, Horacio Capote, Michelle Rainka, Douglas W. Scharre, Maria Kataki, Anahita Adeli, Earl A. Zimmerman, Dzintra Celmins, Alice D. Brown, Godfrey D. Pearlson, Karen Blank, Karen Anderson, Robert B. Santulli, Tamar J. Kitzmiller, Eben S. Schwartz, Kaycee M. Sink, Jeff D. Williamson, Pradeep Garg, Franklin Watkins, Brian R. Ott, Henry Querfurth, Geoffrey Tremont, Stephen Salloway, Paul Malloy, Stephen Correia, Howard J. Rosen, Bruce L. Miller, Jacobo Mintzer, Kenneth Spicer, David Bachman, Elizabether Finger, Stephen Pasternak, Irina Rachinsky, Dick Drost, Nunzio Pomara, Raymundo Hernando, Antero Sarrael, Susan K. Schultz, Laura L. Boles Ponto, Hyungsub Shim, Karen Elizabeth Smith, Norman Relkin, Gloria Chaing, Lisa Raudin, Amanda Smith, Kristin Fargher, Balebail Ashok Raj, Thomas Neylan, Jordan Grafman, Melissa Davis, Rosemary Morrison, Jacqueline Hayes, Shannon Finley, Karl Friedl, Debra Fleischman, Konstantinos Arfanakis, Olga James, Dino Massoglia, J. Jay Fruehling, Sandra Harding, Elaine R. Peskind, Eric C. Petrie, Gail Li, Jerome A. Yesavage, Joy L. Taylor, Ansgar J. Furst

**Affiliations:** 1 Oxidation Biology Unit, The Florey Institute for Neuroscience and Mental Health, The University of Melbourne, Parkville, Victoria 3052, Australia; 2 Bioinformatics Core, The Florey Institute for Neuroscience and Mental Health, The University of Melbourne, Parkville, Victoria 3052, Australia; 3 Cooperative Research Center for Mental Health, Parkville, Victoria 3052, Australia; 4 UC San Francisco; 5 UC San Diego; 6 Mayo Clinic, Rochester; 7 UC Berkeley; 8 U Pennsylvania; 9 USC; 10 UC Davis; 11 Brigham and Women's Hospital/Harvard Medical School; 12 Indiana University; 13 Washington University St. Louis; 14 Prevent Alzheimer's Disease 2020; 15 Siemens; 16 University of Pittsburg; 17 Cornell University; 18 Albert Einstein College of Medicine of Yeshiva University; 19 AD Drug Discovery Foundation; 20 Acumen Pharmaceuticals; 21 Northwestern University; 22 National Institute of Mental Health; 23 Brown University; 24 Eli Lilly; 25 University of Washington; 26 University of London; 27 UCLA; 28 University of Michigan; 29 University of Utah; 30 Banner Alzheimer's Institute; 31 UC Irvine; 32 National Institute on Aging; 33 Johns Hopkins University; 34 Richard Frank Consulting; 35 Oregon Health and Science University; 36 Baylor College of Medicine; 37 University of Alabama – Birmingham; 38 Mount Sinai School of Medicine; 39 Rush University Medical Center; 40 Wien Center; 41 New York University; 42 Duke University Medical Center; 43 University of Kentucky; 44 University of Rochester Medical Center; 45 University of Texas Southwestern Medical School; 46 Emory University; 47 University of Kansas, Medical Center; 48 Mayo Clinic, Jacksonville; 49 Yale University School of Medicine; 50 McGill University/Montreal-Jewish General Hospital; 51 Sunnybrook Health Sciences, Ontario; 52 U.B.C. Clinic for AD & Related Disorders; 53 Cognitive Neurology - St. Joseph's, Ontario; 54 Cleveland Clinic Lou Ruvo Center for Brain Health; 55 Premiere Research Institute, Palm Beach Neurology; 56 Georgetown University Medical Center; 57 Banner Sun Health Research Institute; 58 Boston University; 59 Howard University; 60 Case Western Reserve University; 61 Neurological Care of CNY; 62 Parkwood Hospital; 63 University of Wisconsin; 64 Dent Neurologic Institute; 65 Ohio State University; 66 Albany Medical College; 67 Hartford Hospital, Olin Neuropsychiatry Research Center; 68 Dartmouth-Hitchcock Medical Center; 69 Wake Forest University Health Sciences; 70 Rhode Island Hospital; 71 Butler Hospital; 72 Medical University South Carolina; 73 St. Joseph's Health Care; 74 Nathan Kline Institute; 75 University of Iowa College of Medicine; 76 University of South Florida: USF Health Byrd Alzheimer's Institute; 77 Department of Defence; 78 Stanford University; 79 A full list of members and their affiliations appear at the end of the paper.

## Abstract

Brain iron elevation is implicated in Alzheimer's disease (AD) pathogenesis, but the impact of iron on disease outcomes has not been previously explored in a longitudinal study. Ferritin is the major iron storage protein of the body; by using cerebrospinal fluid (CSF) levels of ferritin as an index, we explored whether brain iron status impacts longitudinal outcomes in the Alzheimer's Disease Neuroimaging Initiative (ADNI) cohort. We show that baseline CSF ferritin levels were negatively associated with cognitive performance over 7 years in 91 cognitively normal, 144 mild cognitive impairment (MCI) and 67 AD subjects, and predicted MCI conversion to AD. Ferritin was strongly associated with CSF apolipoprotein E levels and was elevated by the Alzheimer's risk allele, *APOE-ɛ4*. These findings reveal that elevated brain iron adversely impacts on AD progression, and introduce brain iron elevation as a possible mechanism for *APOE-ɛ4* being the major genetic risk factor for AD.

## Abstract (web summary)

Brain-iron elevation is implicated in Alzheimer's disease (AD), but the impact of the metal on disease outcomes has not been analysed in a longitudinal study. Here, the authors examine the association between the levels of ferritin, an iron storage protein, in the cerebrospinal fluid (CSF) of AD patients and show that CSF ferritin levels predict AD outcomes.

## Graphical abstract

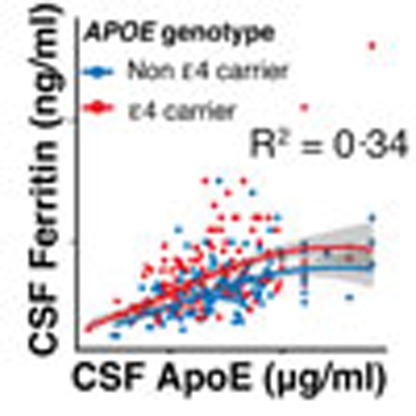

Cortical iron elevation is increasingly reported as a feature of Alzheimer's disease (AD)^1^, and might contribute to the oxidative damage observed in AD brains^2^. A single-blind, 2-year trial of 48 AD patients with the iron chelator, deferoxamine, reported decreased cognitive decline^3^, but this has not been followed up. While evidence in animal models argue in favour of brain iron accumulation propelling atrophy and dementia^4^, prospective evidence about the link between brain iron status and clinical outcomes in AD is lacking.

CSF ferritin could be an index of brain iron load. Ferritin is the iron storage protein of the body and is elevated in AD brain tissue^5^,^6^,^7^,^8^. In cultured systems, ferritin expression^9^,^10^ and secretion^11^ by glia is dependent on cellular iron levels. Ferritin levels in CSF likely reflect iron levels in the brain and can have clinical utility. For example, in Restless Legs Syndrome, a disorder of low brain iron that is treated with iron supplementation, CSF ferritin levels are decreased^12^. CSF ferritin was reported to be elevated in AD in one study^13^, but this was not repeated in subsequent studies using larger clinical cohorts^14^,^15^.

Here, we examined the association of baseline CSF-ferritin data with biomarker, cognitive, anatomical and diagnostic outcomes over 7 years in the Alzheimer's disease Neuroimaging Initiative (ADNI) prospective clinical cohort. We show that CSF ferritin levels have similar utility compared with more established AD CSF biomarkers, the tau/Aβ_1–42_ ratio and apolipoprotein E (ApoE) levels, in predicting various outcomes of AD. However, the nature of the relationship between CSF ferritin levels and cognitive performance was different from the other biomarkers, and, in contrast, CSF ferritin appears as a trait variable, and not a marker of disease.

## Results

### The relationship between CSF ferritin and biomarkers of AD

**Figure 1:**
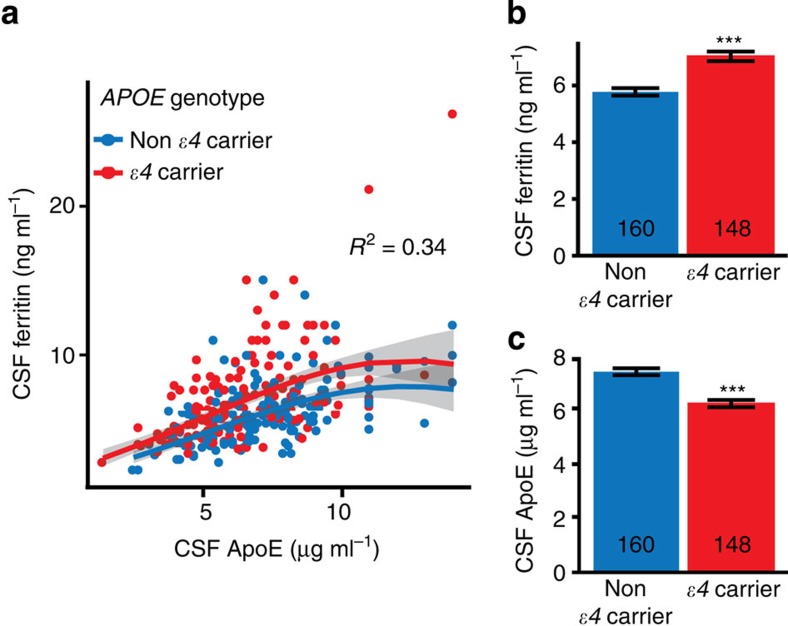
CSF ferritin associates with ApoE levels and varies according to *APOE* genotype. (**a**,**b**) Modelling ferritin in CSF (refer to, M3 of Supplementary Table 1). Minimal multiple regression contained CSF ApoE and *APOE ɛ4*. (**a**) Scatterplot of CSF ApoE and ferritin levels in *APOE ɛ4* carriers and non-*ɛ4* carriers. The genotype did not affect the relationship between ApoE and ferritin; however, genotype is associated with CSF ferritin levels, and thus *ɛ4* carriers and non-*ɛ4* carriers are plotted separately. The *R*^2^ for the linear component of the full model was 0.341 (displayed on graph). (**b**) CSF Ferritin levels in *APOE ɛ4* carriers and non-carriers (ANCOVA: *P*-value=1.10 × 10^−8^). (**c**) Multiple regression of CSF ApoE. ApoE levels in *APOE ɛ4* carriers and non-carriers (ANCOVA: *P*=2.50 × 10^−9^). Data are means+s.e. ‘*n*' is represented in graph columns.

In agreement with other reports^14^,^15^, CSF ferritin levels were not different between cognitively normal (CN; *n*=91), mild cognitive impairment (MCI; *n*=144) and AD (*n*=67) subjects (ANCOVA: *P*=0.591; Table 1) in the ADNI cohort. Neither were there changes in ferritin levels when we separated the cohort according to CSF Aβ_1–42_ levels (192 ng l^−1^ cutoff; as proposed previously^16^) to reflect likely cerebral amyloid burden (ANCOVA: *P*=0.946; Supplementary Fig. 1). But in multiple regression modelling of ferritin including the established CSF biomarkers of AD^17^ (tau, p-tau, Aβ_1–42_), CSF ferritin levels were predicted by Aβ_1–42_ (partial *R*^2^=0.013, *P*=0.029) and tau (partial *R*^2^=0.086, *P*<0.001; model 1, Supplementary Table 1), although not by p-tau. Since the apolipoprotein E gene (*APOE*) alleles are the major genetic risk for AD^18^ and CSF apolipoprotein E protein (ApoE) levels are associated with Aβ_1-42_ (refs 19, 20) and tau^20^,^21^, we re-built the model to include CSF ApoE levels. This abolished the relationship between ferritin and the other biomarkers (Aβ_1–42_: *R*^2^<0.001, *P*=0.904; tau: *R*^2^=0.003, *P*=0.219; model 2, Supplementary Table 1). This led us to detect a surprisingly strong relationship between ApoE and ferritin (linear term partial *R*^2^=0.243, *P*=7.69 × 10^−22^), which was improved when Aβ_1–42_ and tau (non-significant terms) were removed from the model (linear term partial *R*^2^=0.341, *P*=1.52 × 10^−29^; model 3, Supplementary Table 1, Fig. 1a).

In model 3, *APOE* genotype strongly influenced CSF ferritin (*P*=1.10 × 10^−8^), with the major AD risk allele, *ɛ4*, inducing 22% higher levels than non-*ɛ4* carriers (Fig. 1b). Reciprocally, in multiple regression modelling of CSF ApoE, *APOE ɛ4*-positive subjects had lower ApoE levels (-16%; *P*=2.50 × 10^−9^) compared with non-*ɛ4* carriers (Fig. 1c). Plasma ferritin levels were not associated with plasma ApoE levels or *APOE ɛ4* allele status (Supplementary Fig. 2), but there was a modest association between plasma ferritin and CSF ferritin levels (*β*=0.075, *P*=0.0002; Supplementary Fig. 3).

### Association of ferritin with neuropsychiatric assessments

**Figure 2:**
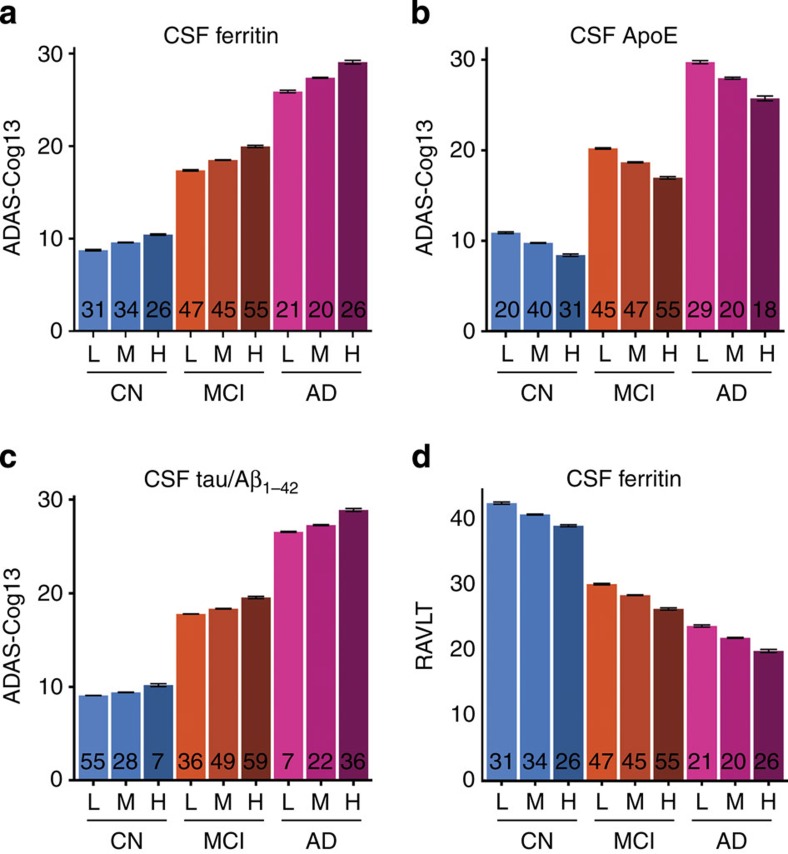
CSF ferritin levels independently predict cognitive status. (**a**–**c**) Multiple regression of baseline ADAS-Cog13 score expressed as tertiles of CSF (**a**) ferritin (L<5.5; H>7.3 ng ml^−1^), (**b**) ApoE (L< 5.8; H>7.8 μg ml^−1^) and (**c**) tau/Aβ_1–42_ (L<0.35; H>0.76). (**d**) Multiple regression of baseline RAVLT score expressed as CSF ferritin tertiles. Data are adjusted for baseline diagnosis, gender, years of education and the AD CSF biomarkers in the minimal models. Data are means+s.e. ‘*n*' is shown in graph columns. CN, cognitively normal; MCI, mild cognitive impairment.

Next, we explored whether CSF ferritin was related to cognitive performance in AD. Baseline ADAS-Cog13 (The Alzheimer's Disease Assessment Scale) score was associated with CSF ferritin (*P*=0.006; Table 2), ApoE levels (*P*=0.0003) and tau/Aβ_1–42_ ratio (*P*=0.025), independently, in a multiple regression model containing the AD biomarkers and other clinical variables. In tertile analysis, high (>7.2 ng ml^−1^), compared with low (<5.4 ng ml^−1^), levels of ferritin were associated with an ≈3 point poorer ADAS-cog13 score (Fig. 2a). Similarly, in tertiles, lower levels of ApoE (Fig. 2b) were associated with a ≈4 point worse ADAS-Cog13, and higher tau/Aβ_1-42_ ratio was associated with a ≈2 point worse ADAS-Cog13 (Fig. 2c), as previously reported^21^,^22^.

To determine whether baseline values of CSF ferritin predict longitudinal cognitive outcome, we constructed a mixed effects model of annual ADAS-Cog13 scores over 7 years (Table 2 for statistics, Supplementary Table 2 for patient numbers) and observed that both ApoE (*P*=0.006) and tau/Aβ_1-42_ ratio (*P*=2.70 × 10^−7^) were still associated with rate of cognitive change (interacted with time), as previously reported^21^,^22^. Ferritin, however, impacted on ADAS-Cog13 by a constant cross-sectional decrement (*P*=4.93 × 10^−4^ main effect only; Table 2).

We additionally modelled cognition using the Rey verbal learning test (RAVLT), which is more sensitive in distinguishing control and MCI patients^23^. In this model, only ferritin levels were associated with cross-sectional cognitive performance (*P*=0.0017; Table 2, Fig. 2d), but CSF ferritin was not associated with rate of deterioration in a longitudinal model (*P*=0.817; Table 2). Baseline tau/Aβ_1–42_ ratio was associated with rate of cognitive decline on RAVLT (*P*=4.8510 × 10^−5^), but there was only a trend for ApoE (*P*=0.066). Hence, in both cognitive scales, CSF ferritin impacted on performance by a constant amount, regardless of disease status, thus appearing as a trait variable and not a marker of disease.

**Figure 3:**
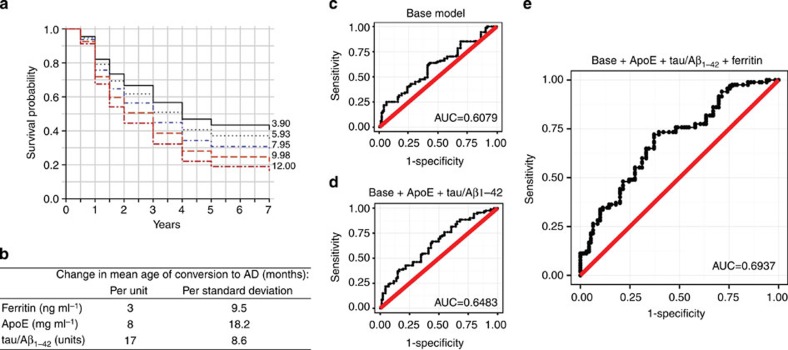
Conversion from MCI to dementia as predicted by baseline CSF biomarkers. (**a**) MCI survival based on the minimal Cox proportional hazards model (Table 2), the conversion is plotted for each quintile of ferritin (applying mean values for the cohort: ApoE=7.2 μg ml^−1^, tau/Aβ_1–42_=0.69 units). The numbers on the right side of the graphs indicate the quintile boundaries. This minimal model contained only the CSF biomarkers. (**b**) Change in mean age of diagnosis according to CSF biomarkers. The months taken for ∼50% survival of each quintile boundary in the Cox models were graphed against the unit values of those boundaries. The gradient of the linear model was used to estimate change in age of conversion for each unit change in analyte. (refer to Fig. 3a, Supplementary Fig. 4). (**c**–**e**) Receiver operating curves of logistic regression modelling of MCI conversion to AD (refer to Table 2, Supplementary Fig. 5). (**c**) Base model controlling for age, gender, BMI, years of education and *APOE ɛ4* status. (**d**) Base model plus ApoE and tau/Aβ_1-42_. (**e**) Base model plus ApoE, tau/Aβ_1–42_ and ferritin. AUC, area under curve.

We reasoned that if high ferritin levels worsened the cognitive performance by a constant value over time, then MCI individuals with high ferritin levels would satisfy the criteria for an AD diagnosis at a comparatively earlier interval. To investigate this, we employed a Cox proportional hazards model on 144 MCI subjects who had CSF ferritin, ApoE and tau/Aβ_1-42_ measurements. In a minimal model (containing only these CSF biomarkers; Table 2) of MCI conversion over 7 years, ferritin (*P*=0.03; Fig. 3a), ApoE (*P*=0.008; Supplementary Fig. 4a) and tau/Aβ_1–42_ (*P*=0.037; Supplementary Fig. 4b) were each significant predictive variables.

Using this model we estimated how many months was required for 50% survivorship for each quintile of each biomarker. We then constructed a linear model of these values (in months; *y*-axis) against the values for the quintile boundaries of each analyte (in designated units; *x*-axis). The gradient of these functions estimates the change in mean age of conversion (in months) associated with one unit change in the baseline CSF analyte. For comparison between biomarkers, we also expressed the change in mean age of conversion associated with a s.d. change to the analyte value. One s.d. change to ferritin was associated with a 9.5-month shift in age of conversion, compared with 18.2 and 8.6 months for ApoE and tau/Aβ_1–42_, respectively (Fig. 3b).

In separate adjusted logistic regression models, an increase in the baseline concentration of each biomarker by its interquartile range increased the odds of converting to AD for ferritin (OR: 1.36, 95%CI: 1.17–1.58) and tau/Aβ_1–42_ ratio (OR: 1.13, CI: 0.95–1.35), and decreased the odds for ApoE (OR: 0.72, CI: 0.61–0.85). Including all three analytes into the one model increased the predictive value of each analyte (OR (CI): ferritin=2.32 (1.86–2.9], tau/Aβ_1–42_=1.45[1.16–1.8], ApoE=0.38[0.3–0.48]; Table 2).

Receiver-operating curves based on the logistic regression models determined the accuracy of these analytes to predict conversion to AD. The area under the curve (AUC) of the base model (age, gender, years of education, BMI, *APOE ɛ4* genotype) was 0.6079 (Fig. 3c), which was increased by the singular inclusions of either ferritin (AUC: 0.6321; Supplementary Fig. 5b), ApoE (0.6311; Supplementary Fig. 5c) or marginally by tau/Aβ_1–42_ (0.6177; Supplementary Fig. 5d). When the tau/Aβ_1–42_ was included in the model containing ApoE, the AUC increased slightly (from 0.6311 to 0.6483; Fig. 3d). This performance, which combined the established CSF biomarkers for AD, was improved markedly by adding ferritin values (from 0.6483 to 0.6937 Fig. 3e).

### Association of ferritin with brain atrophy

**Figure 4:**
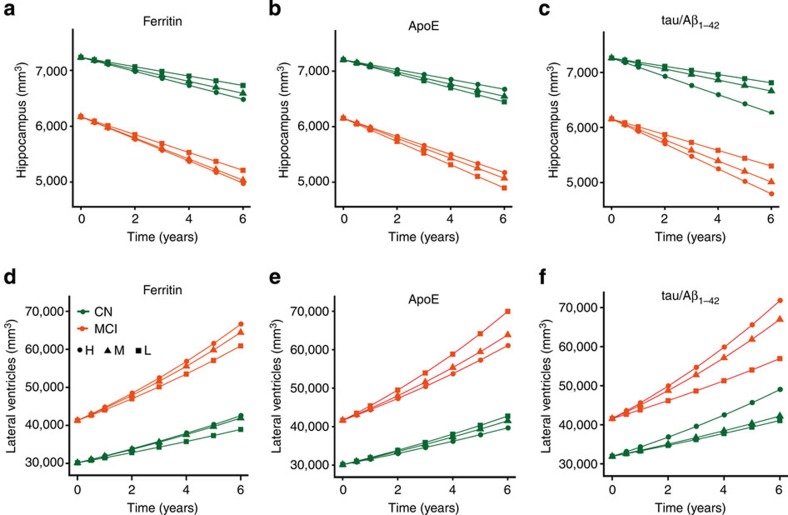
CSF ferritin levels independently predict brain structural changes. (**a**–**c**) Longitudinal hippocampal changes based on tertiles of CSF (**a**) ferritin (L<5.5; H>7.3 ng ml^−1^) (**b**) ApoE (L< 5.8; H>7.8 μg ml^−1^) and (**c**) tau/Aβ_1–42_ (L<0.35; H>0.76) tertiles (refer to Table 2). (**d**–**f**) Longitudinal lateral ventricular changes based on CSF (**d**) ferritin (**e**) ApoE and (**f**) tau/Aβ_1–42_ tertiles (refer to Table 2). These mixed effects models were adjusted for age, gender, baseline diagnosis, years of education, APOE *ɛ4* status and intracranial volume. Tertiles at baseline were not significantly different for all models, therefore for visual display the baseline values were held at the adjusted means for each diagnostic group. CN, cognitively normal; H, highest tertile; M, middle tertile; MCI, mild cognitive impairment; L, lowest tertile.

Finally, we investigated whether ferritin levels associate with neuroanatomical changes to the hippocampus and lateral ventricular area in yearly intervals over a 6-year period for CN and MCI subjects (Supplementary Table 3 for patient numbers). We explored the impact of CSF ferritin when the other biomarkers were also included in modelling, whereas CSF ferritin has previously been shown to predict atrophy of various brain structures when considered in isolation^24^. Baseline ApoE, ferritin and tau/Aβ_1–42_ values each independently predicted hippocampal volume in an adjusted longitudinal model (Table 2). The rate of atrophy of the hippocampus was greater in individuals with high CSF ferritin (*P*=0.02; Fig. 4a). Low CSF ApoE (*P*=0.008; Fig. 4b) or high tau/Aβ_1–42_ (*P*=6.80 × 10^−6^; Fig. 4c) also predicted atrophy, as previous reported^21^,^25^. Lateral ventricular enlargement over time was similarly associated independently with high-CSF ferritin (*P*=0.008; Fig. 4d), low-CSF ApoE (*P*=0.0002; Fig. 4e), or high tau/Aβ_1–42_ (*P*=4.19 × 10^−8^; Fig. 4f).

## Discussion

**Figure 5:**
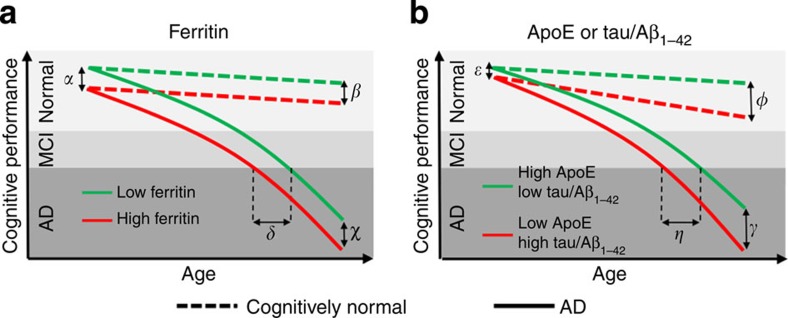
Schematic: the impact of ferritin and other biomarkers on AD presentation. (**a**) CSF ferritin has a qualitatively different impact to (**b**) CSF tau/Aβ_1–42_ and ApoE on cognitive performance over time in cognitively normal (dotted lines) and in subjects who develop AD (solid lines). Higher CSF ferritin levels are associated with poorer baseline cognitive status (for example, RAVLT) by [α] points, where [α]=Ln[ferritin (ng ml^−1^)]*1·77 (refer to Table 2). This effect is constant over time, such that [α]=[β,χ]. Consequently, ferritin causes a shift to the left in age of conversion to AD by [δ] months, where [δ]=ferritin (ng ml^−1^)*3 (refer to Fig. 3b). Levels of tau/Aβ_1–42_ or ApoE are associated with both baseline cognitive status [ɛ] and the rate of cognitive deterioration, such that [ɛ]<[φ,γ]. The effect causes a shift in age of diagnosis by [η] months where [η]=ApoE (μg ml^−1^)*8 or tau/Aβ_1–42_ (units)*17 (refer to Fig. 3b).

Our analyses show that CSF ferritin levels were independently related to cognitive performance in the ADNI cohort and predicted MCI conversion to AD. The magnitude impact of ferritin on these outcomes was comparable to the established biomarkers, ApoE and tau/Aβ_1–42_; however, the nature of the effect of ferritin was not the same. Ferritin was associated with constant shift in cognitive performance over the study period (Fig. 5a), whereas the decrements associated with the other biomarkers were exaggerated over time (Fig. 5b). A downward shift (poorer cognitive presentation) in response to high ferritin levels (1.77 RAVLT points per 1 ng ml^−1^ ferritin; Table 2) results in an earlier age of diagnosis (3 months per 1 ngng ml^−1^ ferritin; Fig. 3b). This would be consistent with findings that patients with an early age of AD onset have greater neocortical iron burden than late-onset patients^1^,^7^. Collectively these data support consideration of therapeutic strategies that lower brain iron, which have reported beneficial outcomes in Phase II trials of Alzheimer's^3^ and Parkinson's^26^ diseases. Lowering CSF ferritin, as might be expected from a drug like deferiprone^26^, could conceivably delay MCI conversion to AD by as much as 3 years.

An unresolved question arising from this study is why are CSF ferritin levels not elevated in AD, where brain iron levels are reported as elevated^2^? We hypothesize that ferritin levels in the CSF reflect global brain iron burden, whereas iron elevation in AD has only been reported in affected regions (for example, frontal cortical tissue^27^). Possibly, iron elevation in brain regions affected by AD is too confined regionally to be reflected in CSF. An altered relationship between tissue and CSF ferritin in AD, however, cannot yet be excluded.

Our data also provide exploratory insights into iron in AD aetiopathogenesis, identifying an unexpected interaction of ApoE with ferritin. That ferritin levels are increased by the *APOE*-*ɛ4* allele argues that ApoE influences ferritin levels, rather than the reverse. Our current findings indicate that *APOE* genotype should influence constitutive brain iron burden. However, to our knowledge, a post mortem study of iron or ferritin in brain tissue, stratified according to *APOE* genotype, has not been reported. Focal changes to iron and ferritin have been observed in AD brains post mortem^1^,^2^,^5^,^6^,^7^,^8^, and on the basis of our findings we propose that the *ɛ4* genotype raises the baseline iron load of the brain, thus lowering the threshold for iron-mediated neuronal loss in disease. This proposal awaits experimental confirmation, but it is possible that increased plaque pathology associated with the *APOE ɛ4* isoform^28^ might be a consequence of interactions between Aβ and iron^29^, leading to oxidative stress and Aβ aggregation^12^,^13^. Elevated iron could likewise contribute to tau pathology by causing its aggregation^30^, indeed iron is co-localized in neurofibrillary tangles in AD, and such co-localization is also observed in a primary disease of brain iron overload, neurodegeneration with brain iron accumulation^2^,^31^. Superficial CNS siderosis is also characterized by brain iron deposition, and tau is elevated in the CSF in this condition^32^. A relationship between brain iron and tau is supported by the results in our study, where CSF ferritin levels correlated with tau levels (Model 1 of Supplementary Table 1; when ApoE is excluded from the analysis).

How, then, could ApoE impact on brain iron homeostasis? To our knowledge, no previous study has directly explored this, but synaptic zinc was reported to be lowered in *APOE* KO mice,^33^ and in a closed head injury model, iron accumulation was shown to be exaggerated in *APOE*-KO mice^34^. The mechanism for ApoE in iron regulation could involve the trafficking of lipoproteins by ApoE. Treatment of macrophages with high-density lipoprotein (HDL; the lipoprotein of CSF) has been shown to lower intracellular iron levels and to promote ferritin release^35^. *APOE ɛ4* carriers have less CSF ApoE, and the *ɛ4* isoform has comparatively lower affinity for HDL^36^, so reduced delivery of HDL in *APOE ɛ4* carriers could result in iron retention in the brain. Notably, the iron accumulation mutation of *HFE* (associated with hemochromatosis) has an epistatic interaction with *APOE ɛ4* to increase AD risk and accelerates disease onset by 5.5 years (reviewed in ref. 37). We therefore introduce the concept that *APOE ɛ4* status confers susceptibility to AD by increasing ferritin levels. The association between ApoE and brain iron status will warrant further investigation.

## Methods

### ADNI description

Data used in the preparation of this article were downloaded on 15 July 2014 from the Alzheimer's Disease Neuroimaging Initiative (ADNI) database (adni.loni.usc.edu). The ADNI study has been previously described in detail^38^. The ADNI was launched in 2003 by the National Institute on Aging (NIA), the National Institute of Biomedical Imaging and Bioengineering (NIBIB), the Food and Drug Administration (FDA), private pharmaceutical companies and non-profit organizations, as a $60 million, five-year public-private partnership. The primary goal of ADNI has been to test whether serial magnetic resonance imaging (MRI), positron emission tomography (PET), other biological markers, and clinical and neuropsychological assessment can be combined to measure the progression of mild cognitive impairment (MCI) and early Alzheimer's disease (AD). Determination of sensitive and specific markers of very early AD progression is intended to aid researchers and clinicians to develop new treatments and monitor their effectiveness, as well as lessen the time and cost of clinical trials.

The principal investigator of this initiative is Michael W. Weiner, MD, VA Medical Center and University of California, San Francisco. ADNI is the result of efforts of many co-investigators from a broad range of academic institutions and private corporations, and subjects have been recruited from over 50 sites across the United States and Canada. The initial goal of ADNI was to recruit 800 subjects but ADNI has been followed by ADNI-GO and ADNI-2. To date these three protocols have recruited over 1,500 adults, ages 55 to 90, to participate in the research, consisting of cognitively normal older individuals, people with early or late MCI, and people with early AD. The follow-up duration of each group is specified in the protocols for ADNI-1, ADNI-2 and ADNI-GO. Subjects originally recruited for ADNI-1 and ADNI-GO had the option to be followed in ADNI-2. For up-to-date information, see www.adni-info.org.

### Recruitment inclusion and exclusion criteria for ADNI 1

Inclusion criteria were as follows: (1) Hachinski Ischaemic Score ≤4; (2) permitted medications stable for 4 weeks before screening; (3) Geriatric Depression Scale score<6; (4) visual and auditory acuity adequate for neuropsychological testing; good general health with no diseases precluding enrolment; (5) six grades of education or work history equivalent; (6) ability to speak English or Spanish fluently; (7) a study partner with 10 h per week of contact either in person or on the telephone who could accompany the participant to the clinic visits.

Criteria for the different diagnostic groups are summarized in Supplementary Table 1. Groups were age-matched. Cognitively normal (CN) subjects must have no significant cognitive impairment or impaired activities of daily living. Clinical diagnosed AD patients must have had mild AD and had to meet the National Institute of Neurological and Communicative Disorders and Stroke–Alzheimer's Disease and Related Disorders Association criteria for probable AD^39^, whereas mild cognitive impairment subjects (MCI) could not meet these criteria, have largely intact general cognition as well as functional performance, but meet defined criteria for MCI.

### CSF biomarker collection and analysis

CSF was collected once in a subset of ADNI participants at baseline. Aβ_1–42_ and tau levels in CSF were measured using the Luminex platform. ApoE and ferritin protein levels were determined using a Myriad Rules Based Medicine platform (Human Discovery MAP, v1.0; see ADNI materials and methods). CSF Factor H (FH) levels were measured using a multiplex human neurodegenerative kit (HNDG1-36K; Millipore, Billerica, MA) according to the manufacturer's overnight protocol with minor modifications.

CSF was collected into polypropylene tubes or syringes provided to each site, and then be transferred into polypropylene transfer tubes without any centrifugation step followed by freezing on dry ice within 1 h after collection for subsequent shipment overnight to the ADNI Biomarker Core laboratory at the University of Pennsylvania Medical Center on dry ice. Aliquots (0.5 ml) were prepared from these samples after thawing (1 h) at room temperature and gentle mixing. The aliquots were stored in bar code-labelled polypropylene vials at −80 °C. Fresh, never before thawed, 0.5 ml aliquots for each subject's set of longitudinal time points were analysed on the same 96-well plate in the same analytical run for this study to minimize run to run and reagent kit lot sources of variation. Within run coefficient of variation (%CV) for duplicate samples ranged from 2.5 to 5.9% for Aβ_1–42_, 2.2–6.3% for tau and the inter-run %CV for CSF pool samples ranged from 5.1 to 14% for Aβ_1–42_, 2.7–11.2% for tau.

Apolipoprotein E (ApoE) and ferritin protein levels were determined using Rules Based Medicine (Human Discovery MAP, v1.0).

Further information on the procedures and standard operating procedures can be found in previous publications^40^,^41^ and online ( http://www.adni-info.org/).

### Structural MRI acquisition and processing

Subjects with a 1.5-T MRI and a sagittal volumetric 3D MPRAGE with variable resolution around the target of 1.2 mm isotropically were included in the analysis. See ( www.loni.ucla.edu/ADNI) and for detail^42^. The hippocampal and ventral volumes utilized were those in the ADNIMERGE primary table as part of the ADNIMERGE R package, downloaded on the 15 July 2014. Only CN and MCI subjects were included in the MRI analysis. MRI scans were performed at baseline, 6 months, 1 year and then yearly for six years.

### Statistical analysis

All statistical work was conducted with R (version 3.1.0)^43^, using packages ggplot2 (ref. 44), nlme^45^, car^46^ and Deducer^47^. We tested the conditions necessary to apply the regression models, normal distribution of the residuals and the absence of multicolinearity. All models satisfied these conditions. Minimal models were obtained via step down regression using Akaike information criterion (AIC), and Bayesian information criterion (BIC), ensuring that the central hypotheses were maintained. Subjects were excluded from analysis if they had one or more covariates missing. Where subjects prematurely left the study, their data were included in modelling to the point at which they left. The following variables were natural log-transformed to ensure normality: CSF ferritin, Factor H, tau and haemoglobin, while ADAS-cog13 was square-root transformed.

ANCOVA models assessing the differences in each of the CSF biomarkers across the diagnostic groups initially contained age, gender, BMI, *APOE* genotype, and levels of CSF haemoglobin (Hb) and Factor H (FH). CSF Hb was included as a proxy for blood contamination, to control for the possibility of a traumatic tap introducing plasma ferritin into the CSF samples. FH was used to control for inflammation, since ferritin levels are known to be elevated in certain inflammatory conditions (for example, bacterial meningitis^48^).

Multiple regression models of CSF ferritin and ApoE initially contained age, gender, BMI, *APOE* genotype, and levels of CSF Hb and FH, plus various inclusions of CSF tau, Aβ_1–42_ and either ferritin or ApoE. The minimal models are described in the table legend.

Associations between the baseline Alzheimer's Disease Assessment Scale Cognition (ADAS-cog13) and Rey Auditory Verbal Learning Test (RAVLT) scores with CSF ferritin, the CSF tau/Aβ_1–42_ ratio and CSF ApoE were tested with a covariate-adjusted multiple regression for each cogntive scale. For these analyses, age, gender, BMI, years of education, *APOE*-*ɛ4* allele and baseline diagnosis were initially included as covariates. To assess the association of baseline CSF ferritin levels with the longitudinal clinical outcomes (ADAS-cog13 and RAVLT scores over 7 years), linear mixed effects models were used. These models were adjusted for the same variables as the baseline models of cognition, and additionally included time as interacting variable with each of the CSF biomarkers. AD subjects were excluded from the longitudinal analysis because of low rate of follow up (Supplementary Table 2). A significant value for any of these interaction terms would indicate that the variable affected the rate of cognitive change. For the ADAS-cog13, longitudinal analysis, the minimal model included years of education, gender and *APOE*-*ɛ4* allele. For the longitudinal analysis with RAVLT, the minimal model included years of education and gender.

Cox proportional hazards model was used to assess the impact of CSF analytes on the time to AD conversion. The initial model contained age at baseline, gender, years of education and *APOE*-*ɛ4* genotype as confounding variables together with CSF ApoE, tau/Aβ_1–42_ and ferritin. A minimal model containing only the CSF biomarkers was identified via BIC step down procedure and log likelihood test.

Logistic regression analysis was used to assess the impact of CSF analytes on risk of conversion to AD. Combinations of CSF ferritin, ApoE and tau/Aβ_1–42_ analytes were included in logistic regression models of MCI conversion to AD that were adjusted for age at baseline, gender, years of education, *APOE* genotype and BMI. These models determined the predictive performance of these analytes in identifying stable MCI participants from MCI participants who, up to 102 months, had a diagnosis change to AD. The receiver-operator curves and the area under the curve were derived from the predictive probabilities of the logistic regression models.

The relationships between CSF ferritin, ApoE, tau/Aβ_1–42_ with longitudinal structural (MRI) changes to hippocampus and lateral ventricle were analysed using linear mixed models adjusted for age, years of education, BMI, gender and *APOE* genotype and intracranial volume. For all models, CSF ferritin, ApoE, tau/Aβ_1–42_ and baseline diagnosis were included as fixed effects and were not removed from a minimal model. Two random effects were also included, intercepts and slope (time). An interaction between time and diagnosis, time and CSF ferritin, time and CSF ApoE, as well as time and CSF tau/Aβ_1–42_ were also included for all models. All the AD subjects were excluded from MRI analyses due to low numbers and short follow-up. PET imaging data from ADNI were not included in the analysis because there were too few patients who had CSF ferritin measured and who also underwent PET imaging at baseline.

## Additional information

**How to cite this article:** Ayton, S. *et al*. Ferritin levels in the cerebrospinal fluid predict Alzheimer's disease outcomes and is regulated by APOE. *Nat. Commun.* 6:6760 doi: 10.1038/ncomms7760 (2015).

## Supplementary Material

Supplementary Information Supplementary Figures 1-5 and Supplementary Tables 1-3

## Figures and Tables

**Table 1:** Baseline characteristics of subjects from the ADNI cohort used in this study, stratified by diagnosis.

	**Units**	**CN**	**MCI**	**AD**	**P-value**
n	—	91	144	67	NA
Age	Years (s.d.)	75.74 (5.43)	74.85 (7.2)	74.57 (7.61)	0.502
Female	*n* (%)	46 (50.55)	47 (32.64)	29 (43.28)	*0*.*021*
Education	Years (s.d.)	15.67 (2.94)	15.91 (2.95)	15.01 (2.96)	0.123
*APOE*-ɛ4 +ve	*n* (%)	22 (24.18)	75 (52.08)	46 (68.66)	*6.50 × 10*^*−8*^
ADAS-Cog13	Units (s.d.)	9.51 (4.16)	19.19 (5.94)	29.22 (8.21)	*2.75 × 10*^*−56*^
CSF Ferritin	ng ml^−1^ (s.d.)	6.4 (2.07)	6.95 (2.72)	6.94 (2.99)	0.591
CSF ApoE	μg ml^−1^ (s.d.)	7.3 (2.21)	7.1 (2.22)	6.35 (2.27)	*0.012*
CSF tau	pg ml^−1^ (s.d.)	69.78 (28.01)	104.3 (52.41)	122.63 (57.47)	*4.57 × 10*^*−7*^
CSF ptau	pg ml^−1^ (s.d.)	24.77 (13.34)	36.39 (16.09)	41.39 (20.76)	*1.13 × 10*^*−6*^
CSF Aβ_1-42_	pg ml^−1^ (s.d.)	205.31 (56.38)	161.06 (52.06)	142.16 (36.84)	*2.29 × 10*^*−6*^
CSF tau/Aβ_1–42_	Units (s.d.)	0.39 (0.26)	0.75 (0.5)	0.94 (0.52)	*7*.*80 × 10*^*−9*^
Hippocampus	mm^3^ (s.d.)	7,219.6 (848.6)	6,230.9 (1,047.8)	5.766.6 (1,283.2)	*6.71 × 10*^*−20*^
Lateral ventricle	mm^3^ (s.d.)	34,052.62 (16,528.1)	44,842.52 (23,574.05)	49,902.53 (26,896.68)	*3.35 × 10*^*−5*^

AD, Alzheimer's disease; CN, cognitively normal; CSF, cerebrospinal fluid; MCI, mild cognitive impairment. Unadjusted unit values are presented in the table. *P* values presented for ANCOVA models of CSF analytes and MRI brain structure were adjusted for age, gender, years of education, BMI, *APOE* genotype, CSF haemoglobin and CSF Factor H. Intracranial volume was also included in ANCOVA models of brain structure.

**Table 2:** Modelling the association of CSF biomarkers on AD outcomes.

**Model**	Ferritin*		**tau/Aβ**_**1–42**_		**ApoE**	
Cross-sectional cognition (MR)	β (s.e.)	*P* value	β (s.e.)	*P* value	β (s.e.)	*P* value
ADAS-Cog13†	0.139 (0.050)	*0.006*	0.104 (0.046)	*0.025*	−0.178 (0.049)	*0.0003*
RAVLT	−1.77 (0.559)	*0.0017*	NS	NS	1.033 (0.564)	0.0677
						
*Longitudinal cognition (MELM)*	β (s.e.)	*P* value	β (s.e.)	*P* value	β (s.e.)	*P* value
ADAS-Cog13†						
Main effect	0.178 (0.051)	*0.0005*	0.129 (0.049)	*0*.*009*	−0.180 (0.051)	*0*.*0004*
Interaction time	0.0005 (0.016)	0.977	0.081 (0.016)	*2*.*70 × 10*^*−7*^	−0.044 (0.016)	*0*.*006*
RAVLT						
Main effect	−1.60 (0.63)	*0*.*012*	−0.847 (0.608)	0.165	1.03 (0.63)	0.104
Interaction time	−0.035 (0.152)	0.817	−0.610 (0.150)	*4*.*85 × 10*^*−5*^	0.279 (0.152)	0.066
						
MCI conversion to AD	Statistic‡	*P* value	Statistic‡	*P* value	Statistic‡	*P* value
Cox (Hazard ratio)	1.10 (1.01–1.19)	*0.030*	1.53 (1.03–2.28)	*0.037*	0.83 (0.73–0.95)	*0.008*
LR (Odds ratio)	2.32 (1.86–2.90)	*8.001 × 10*^*−15*^	1.45 (1.16–1.80)	*0.0001*	0.38 (0.30–0.48)	*1.88 × 10*^*−17*^
						
Rate of MRI atrophy (MELM)	β (s.e.)	*P* value	β (s.e.)	*P* value	β (s.e.)	*P* value
Hippocampus	−18.33 (7.86)	*0.019*	−35.31 (7.79)	*6.81 × 10*^*−6*^	21.38 (8.02)	*0.008*
Lateral ventricles§	0.007 (0.003)	*0.008*	0.013 (0.002)	4.19 × 10^*−*8^	−0.009 (0.003)	*0.0002*

Cox, Cox proportional hazard model; LR: logistic regression; MELM, mixed effects linear model; MR, multiple regression; NS, not significant. All models initially contained the variables: age, gender, BMI, *APOE* genotype, baseline diagnosis; the MRI models additionally included intracranial volume. Minimal models for the cognition models included baseline diagnosis, gender, years of education and the AD CSF biomarkers. Minimal model for the Cox proportional hazard model (Cox) contained only the AD CSF biomarkers. Minimal models for the MRI models contained age, gender, baseline diagnosis, years of education, *APOE ɛ4* status and intracranial volume. All subjects with available data were included in the cross-sectional cognition models. Only CN and MCI subjects were included in modelling of longitudinal cognition because short follow up of AD subjects (Supplementary Table 3). Only subjects who were classed as MCI at baseline were included in the MCI conversion models. The MRI models contained subjects who were classed as cognitively normal or MCI at baseline. AD subjects at baseline were not included because of low numbers and lack of follow-up (Supplementary Tables 3).

^*^Ferritin values were log-transformed, excluding non-parametric Cox and LR models.

^†^The β-coefficient is for the square root of ADAS-Cog13.

^‡^The statistics for the conversion models were based on one interquartile range change for each analyte (ferritin: 3.3 ng ml^−1^, tau/Aβ_1–42_: 0.67 units; ApoE: 3.1 μg ml^−1^).

^§^For Lateral ventricles, the β-coefficient is for natural log of the ventricle volume.
