# Supplementary material for: Ferritin levels in the cerebrospinal fluid predict Alzheimer's disease outcomes and are regulated by APOE
Source: Nat Commun. 2015 May 19;6:6760. doi: 10.1038/ncomms7760 (PMC4479012; doi:10.1038/ncomms7760)
Supplement: Supplementary Information — Supplementary Figures 1-5 and Supplementary Tables 1-3 [file ncomms7760-s1.pdf]

Supplementary Figures

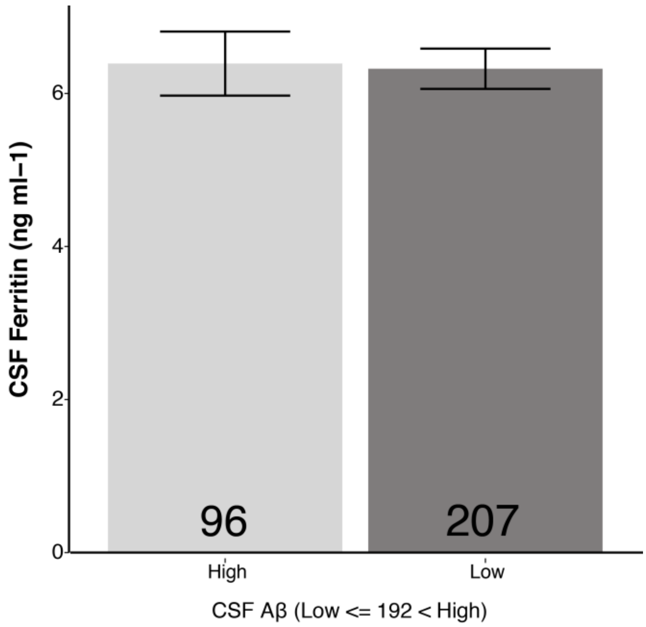

Supplementary Figure 1. CSF ferritin levels in subjects with low (<192 ng/L) and higher (>192 ng/L) CSF Aβ. ANCOVA model (P=0.946) origianlly contained age, gender, BMI, *APOE* ε4 genotype, CSF ApoE levels, CSF Factor H. The minimal model contained BMI, factor H, *APOE* ε4 genotype, CSF ApoE levels. Data means + std error. 'n' in columns.

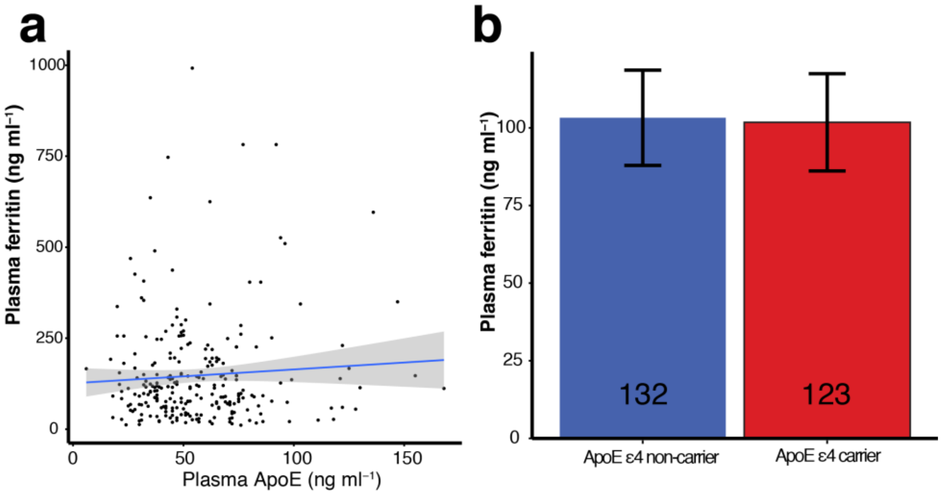

Supplementary Figure 2. No relationship between plasma ferritin and APOE. Data from multiple regression modeling of plasma ferritin initially including age, gender, diagnosis, BMI, *APOE* ε4 genotype, plasma ApoE. Minimal model contained gender, and ApoE and APOE ε4 were retained for analysis. **(a)** Scatter blot of plasma ApoE and plasma ferritin. **(b)** Plasma ferritin levels according to *APOE* ε4 genotype. Data means + std error. 'n' in columns.

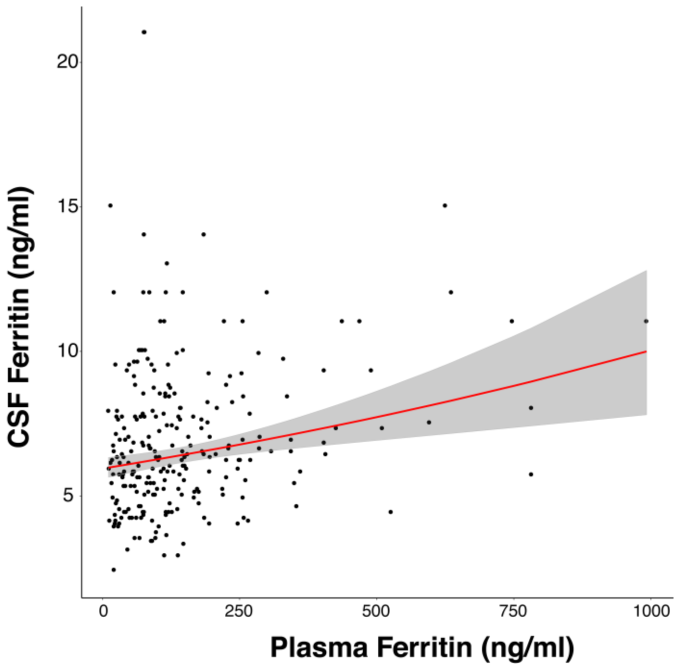

Supplementary Figure 3. Associations between CSF ferritin and plasma ferritin. Scatter plot of multiple regression model initially containing age, gender, CSF Factor H, baseline diagnosis, *APOE* genotype and BMI. The minimal model was adjusted for CSF Factor H, BMI and *APOE* genotype. MR: β=0.075, *p*= 0.0002

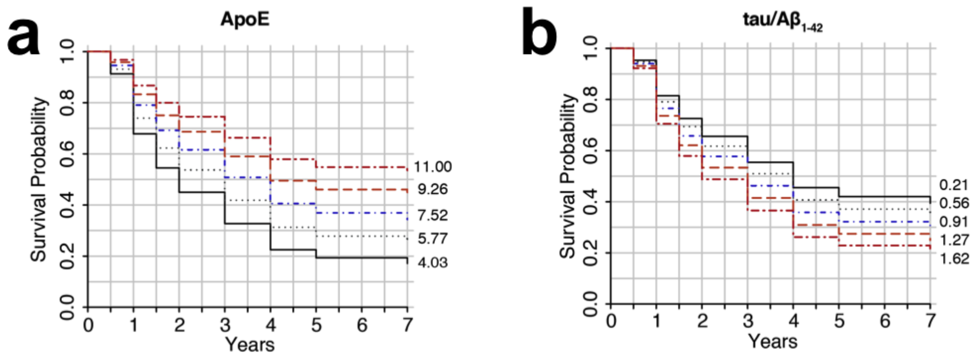

Supplementary Figure 4. Conversion from MCI to dementia as predicted by baseline CSF biomarkers. Based on the minimal Cox proportional hazards model (cf. **Table 1**), the conversion is plotted for each quintile of **(a)** ApoE (ferritin =6·5 ng/mL, tau/Aβ_{1-42} =0·69 units) and **(b)** tau/Aβ_{1-42} (ferritin =6·5 ng/mL, ApoE =7·2 µg/mL). The numbers on the right side of the graphs indicate the quintile boundaries.

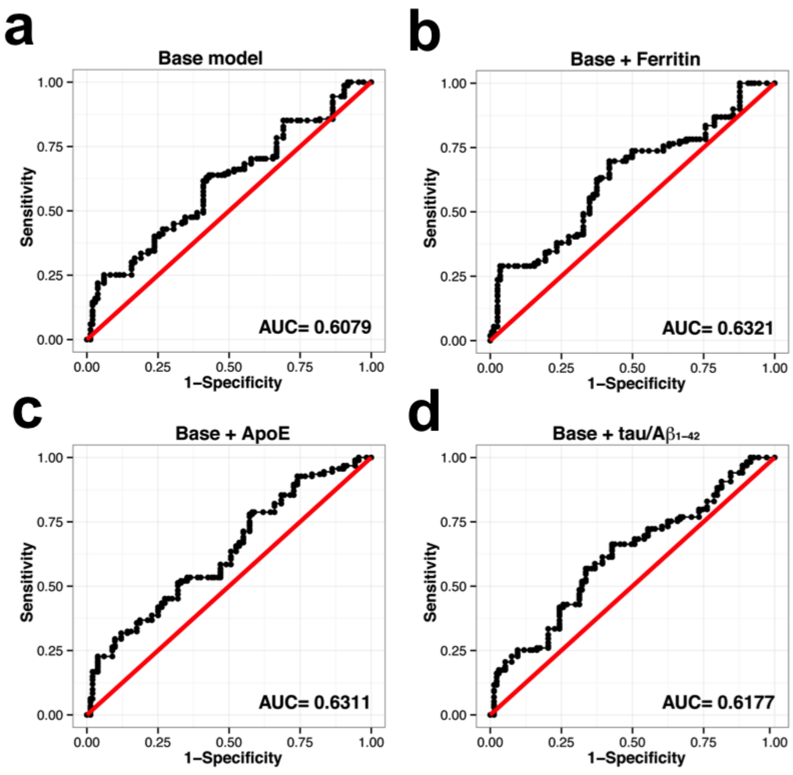

Supplementary Figure 5. Utility of CSF ferritin as a biomarker for MCI conversion to AD. Receiver operating curves of logistic regression modeling of MCI conversion to AD (cf. Table 1). **(a)** Base model containing the demographic information: age, gender, BMI, years of education, and *APOE* ε4 status. **(b)** Base model plus CSF ferritin. **(c)** Base model plus CSF ApoE. **(d)** Base model plus tau/A$\beta_{1\text{-}42}$. AUC- Area Under Curve.

Supplementary Tables

Supplementary Table 1. Modeling of the relationships between CSF ferritin and CSF biomarkers of Alzheimer's disease. Presented are three models to explore the associations between CSF ferritin levels and the two established CSF biomarkers, Aβ1-42 and tau (M1 and M2), as well as the association between CSF ferritin levels and the newer candidate CSF biomarker, ApoE protein level (M2 & M3). All models initially contained the variables: age, gender, BMI, *APOE* genotype, baseline diagnosis, and levels of CSF tau, p-tau, $A\beta_{1\text{-}42}$, Hb and FH. M2 & M3 additionally included ApoE CSF levels. **M1** minimal model contained: *APOE* genotype, tau, BMI, gender, and FH. **M2** minimal model contained: *APOE* genotype and ApoE levels, and tau and $A\beta_{1\text{-}42}$ were retained. **M3** minimal model contained the same as M2, but tau and $A\beta_{1\text{-}42}$ were dropped. AIC- Akaike information criterion, BIC- Bayesian information criterion.

	$A\beta_{1\text{-}42}$			tau			ApoE			$ApoE^2$				
Model	β	pR^2	*p*-value	β	pR^2	*p*-value	β	pR^2	*p*-value	β	pR^2	*p*-value	AIC	BIC
M1	0.051	0·013	*0·029*	0.129	0·086	*4·12x10^{-8}*	-	-	-	-	-	-	160	189·5
M2	0.003	0·000	0·904	0.026	0·003	0·219	0.213	0·236	*7.69x10^{-22}*	0.045	0·028	*0.0004*	95·62	121·4
M3	-	-	-	-	-	-	0.224	0·341	*4.04x10^{-29}*	0.047	0·049	*0.0002*	93·32	111·7

Supplementary Table 2. Patient numbers for longitudinal cognitive assessment. Bl: Baseline. CN: cognitively normal. MCI: Mild cognitive impairment. AD: Alzheimer's disease

	CN	MCI	AD
Bl	88	137	63
6m	88	137	61
1yr	86	138	63
2yr	82	123	52
3yr	78	97	4
4yr	55	47	2
5yr	49	39	0
6yr	54	37	0
7yr	43	27	0

Supplementary Table 3. Patient numbers for longitudinal MRI assessment. Bl: Baseline. CN: cognitively normal. MCI: Mild cognitive impairment. AD: Alzheimer's disease

	CN	MCI	AD
**Bl**	79	108	48
**6m**	80	108	49
**1yr**	74	96	37
**2yr**	66	85	35
**3yr**	57	62	0
**4yr**	38	35	0
**5yr**	26	24	0
**6yr**	24	14	0
